# Molecular Mechanism of *Gelsemium elegans* (Gardner and Champ.) Benth. Against Neuropathic Pain Based on Network Pharmacology and Experimental Evidence

**DOI:** 10.3389/fphar.2021.792932

**Published:** 2022-01-03

**Authors:** Wancai Que, Zhaoyang Wu, Maohua Chen, Binqing Zhang, Chuihuai You, Hailing Lin, Zhichang Zhao, Maobai Liu, Hongqiang Qiu, Yu Cheng

**Affiliations:** ^1^ Department of Pharmacy, Fujian Medical University Union Hospital, Fuzhou, China; ^2^ College of Pharmacy, Fujian Medical University, Fuzhou, China; ^3^ College of Agriculture, Fujian Agriculture and Forestry University, Fuzhou, China; ^4^ Department of Pharmacy, Shengjing Hospital of China Medical University, Shenyang, China

**Keywords:** *Gelsemium elegans* (Gardner and Champ.) Benth, neuropathic pain, network pharmacology, molecular docking, molecular dynamics simulation

## Abstract

*Gelsemium elegans* (Gardner and Champ.) Benth. (Gelsemiaceae*)* (GEB) is a toxic plant indigenous to Southeast Asia especially China, and has long been used as Chinese folk medicine for the treatment of various types of pain, including neuropathic pain (NPP). Nevertheless, limited data are available on the understanding of the interactions between ingredients-targets-pathways. The present study integrated network pharmacology and experimental evidence to decipher molecular mechanisms of GEB against NPP. The candidate ingredients of GEB were collected from the published literature and online databases. Potentially active targets of GEB were predicted using the SwissTargetPrediction database. NPP-associated targets were retrieved from GeneCards, Therapeutic Target database, and DrugBank. Then the protein-protein interaction network was constructed. The DAVID database was applied to Gene Ontology and Kyoto Encyclopedia of Genes and Genome pathway enrichment analysis. Molecular docking was employed to validate the interaction between ingredients and targets. Subsequently, a 50 ns molecular dynamics simulation was performed to analyze the conformational stability of the protein-ligand complex. Furthermore, the potential anti-NPP mechanisms of GEB were evaluated in the rat chronic constriction injury model. A total of 47 alkaloids and 52 core targets were successfully identified for GEB in the treatment of NPP. Functional enrichment analysis showed that GEB was mainly involved in phosphorylation reactions and nitric oxide synthesis processes. It also participated in 73 pathways in the pathogenesis of NPP, including the neuroactive ligand-receptor interaction signaling pathway, calcium signaling pathway, and MAPK signaling pathway. Interestingly, 11-Hydroxyrankinidin well matched the active pockets of crucial targets, such as EGFR, JAK1, and AKT1. The 11-hydroxyrankinidin-EGFR complex was stable throughout the entire molecular dynamics simulation. Besides, the expression of EGFR and JAK1 could be regulated by koumine to achieve the anti-NPP action. These findings revealed the complex network relationship of GEB in the “multi-ingredient, multi-target, multi-pathway” mode, and explained the synergistic regulatory effect of each complex ingredient of GEB based on the holistic view of traditional Chinese medicine. The present study would provide a scientific approach and strategy for further studies of GEB in the treatment of NPP in the future.

## Introduction

Chronic pain condition is a major health issue that comprises five of the 11 top-ranking conditions lived with disability and is responsible for economic burden worldwide (Vos et al., 2012; Andrew et al., 2014). The prevalence of neuropathic pain (NPP) as a feature of chronic pain was estimated to range from 1 to 17.9% (van Hecke et al., 2014). NPP is defined as an injury or disease of the somatosensory system involving complex pathogenesis according to the 2011 International Association for the Study of Pain (Jensen et al., 2011). Overall, the current pharmacological interventions in NPP primarily consist of antidepressants or antiepileptics as the first-line treatments (Lunn et al., 2014; Moore and Gaines, 2019), lidocaine plasters, capsaicin high concentration patches, and tramadol as the second-line treatments (van Nooten et al., 2017; Kim et al., 2018), and strong opioids and botulinum toxin A as the third-line treatments (Sommer et al., 2020). Unfortunately, patients with NPP conventional have an inadequate response with only 40–60% of patients achieving partial relief to the current pharmacological therapy and suffering from side effects include sedation, anticholinergic effects, nausea, and orthostatic hypotension (Dworkin et al., 2007; Cavalli et al., 2019). Therefore, there is a necessity to explore more effective analgesics with novel mechanisms and low side effects for the treatment of NPP.

Traditional Chinese medicine (TCM) is an abundant resource for drug development and provides innovative insight into therapeutic approaches. *Gelsemium elegans* (Gardner and Champ.) Benth. (Gelsemiaceae) (GEB) is a toxic plant indigenous to Southeast Asia especially China, which has long been used as Chinese folk medicine for the treatment of various types of pain, such as neuralgia, sciatica, rheumatoid arthritis, and acute pain (Rujjanawate et al., 2003; Lin et al., 2021). Phytochemical studies have revealed that the main active ingredients of GEB are alkaloids, especially the indole alkaloids, such as koumine, gelsemine, gelsenicine, and gelsevirine (Jin et al., 2014). These alkaloids are distributed throughout the whole plant, especially rich in the roots. GEB and its active alkaloids have been studied increasingly and exert promising pharmacological effects in NPP. It was reported that a crude alkaloidal extract solution from GEB could significantly increase the pain thresholds of mice in both hot plate and writhing tests at the dose of 0.5, 1.0, and 2.0 mg/kg (Rujjanawate et al., 2003). As an important active ingredient, previous studies indicated that koumine exhibited a significant analgesic effect *in vitro* and in several animal models of NPP. These studies suggested that koumine alleviated NPP may through a wide variety of mechanisms, including enhancing 3α-hydroxysteroid oxidoreductase mRNA expression and bioactivity (Qiu et al., 2015) in the spinal cord, upregulating allopregnanolone (Xu et al., 2012), and inhibiting astrocyte activation as well as M1 polarization while sparing the anti-inflammatory responses to NPP (Jin et al., 2018a; Jin et al., 2018b). Other active ingredients, gelsemine, gelsenicine, and gelsevirine may produce antinociception by activating the spinal α3 glycine/allopregnanolone pathway (Zhang and Wang, 2015). However, all the existing studies focused on limited ingredients, targets, and pathways, and lacked the integral thoughts and exploration on TCM with multiple ingredients and targets. Hence, the interactions between ingredients-targets-pathways and other underlying molecular mechanisms of GEB against NPP remain unclear.

**FIGURE 1:**
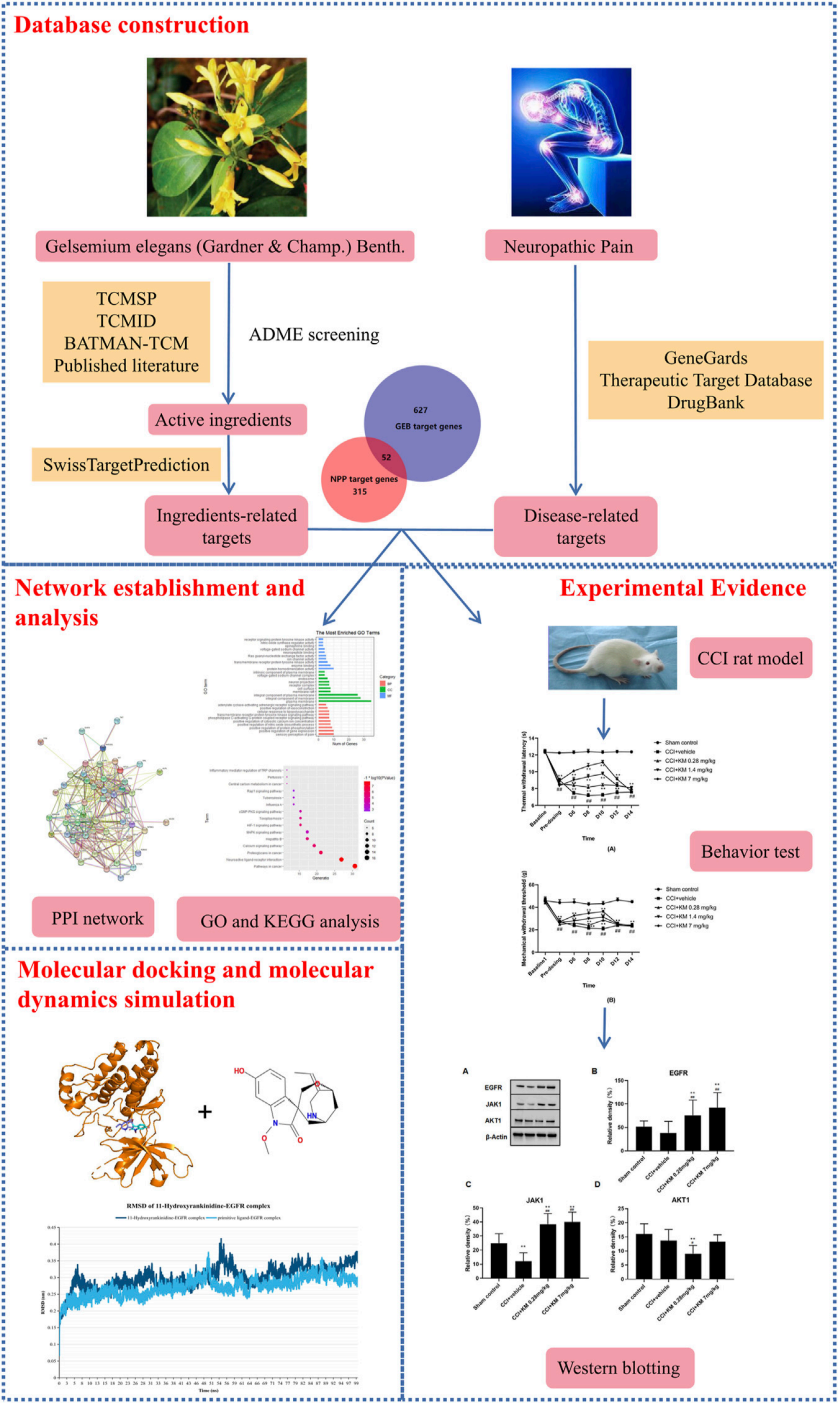
The flowchart of network pharmacology analysis.

Network pharmacology is mostly used to screen the active ingredients, predict the corresponding target, and explore the comprehensive molecular mechanisms of TCM. The key ideas of network pharmacology are based on the theory of system biology and multi-direction pharmacology, which are consistent with the holistic philosophy of TCM (Li and Zhang, 2013). Molecular docking simulation is a computational method for exploring the ligand conformations adopted within the binding sites of receptors in the intermolecular recognition process (Ferreira et al., 2015). Different from traditional pharmacological research methods of TCM, network pharmacology-based analysis combined with molecular docking technology could provide a new perspective for the study of the molecular mechanism of TCM. In the present study, we proposed an “ingredient-target-pathway” network to reveal the potential material basis and compatibility molecular mechanisms of GEB against NPP based on the network pharmacology and experimental evidence. The flowchart of our work is shown in Figure 1.

## Materials and Methods

### Identification of Active Ingredients in GEB

The potential active ingredients in GEB were retrieved from the published literature (Jin et al., 2014) and the online public databases, including the Traditional Chinese Medicines Integrated database (TCMID) (http://www.megabionet.org/tcmid/) (Huang et al., 2018), Bioinformatics Analysis Tool for Molecular mechanism of Traditional Chinese Medicine (BATMAN-TCM) (http://bionet.ncpsb.org/batman-tcm/) (Liu et al., 2016), and Traditional Chinese Medicine database@ Taiwan (http://tcm.cmu.edu.tw/zh-tw/) (Chen, 2011). Active ingredients with oral bioavailability (OB) ≥ 30% and drug-likeness (DL) ≥ two of five features (Lipinski, Ghose, Veber, Egan, and Muegge) were selected, which was recommended by SwissADME (http://www.swissadme.chwebsite) (Daina et al., 2017). The final cluster of chemical ingredients of GEB was determined after removing duplicates.

### Identification of Ingredients-Related Targets

Targets of the active ingredients were predicted using SwissTargetPrediction (http://www.swisstargetprediction.ch), a popular online server that could accurately predict the targets of bioactive molecules with known ligands (Gfeller et al., 2014). 3D structural SDF formats (.sdf) of active ingredients of GEB were acquired from the PubChem database (https://pubchem.ncbi.nlm.nih.gov/) and imported into SwissTargetPrediction for identification of potential drug targets in humans. After removing duplicate targets, the targets of ingredients with SwissTargetPrediction probability ≥0.1 were chosen as potential targets, and compounds without target information were excluded.

### Identification of Disease-Associated Targets

The disease-associated targets of NPP were collected from GeneCards (https://www.genecards.org/) (Safran et al., 2010), the Therapeutic Target database (https://db.idrblab.org/ttd/) (Wang et al., 2020a), and DrugBank (https://go.drugbank.com) (Wishart et al., 2018). “Neuralgias”, “Neuropathic Pain”, “Neurodynia”, and “Nerve Pain” were used as keywords in the three databases and Homo sapiens targets with a disease relevance score ≥ of three were selected for the study.

### Topology Analysis of the Protein-Protein Interaction (PPI) Network

The intersection of ingredients-related targets and disease-associated targets was visualized by overlapping with a Venn diagram. Then, a PPI network was constructed through the String database (https://stringdb.org/) to explore the core regulatory genes (Szklarczyk et al., 2019). PPI information was extracted with an interaction score of 0.4 and the species was only limited to “Homo sapiens”. The topology analysis of the PPI was performed with Cytoscape 3.7.2 (http://cytoscape.org/.ver.3.7.2). NetworkAnalyzer analysis was used to screen key targets according to the degree value. The top 15 important proteins with a higher level of degrees in the interaction network were considered as the key targets for GEB in the treatment for NPP. Furthermore, the Molecular Complex Detection (MCODE) plugin was used to detect cluster modules from the complex network with the node score cutoff of 0.2, K-core of 2, and degree cutoff of 2.

### Gene Ontology (GO) and Kyoto Encyclopedia Genes Genomes (KEGG) Enrichment Analysis

The GO and KEGG enrichment analysis were performed to explore the signaling pathways and bioprocesses involved in the key targets. The database for Annotation, Visualization, and Integrated Discovery (DAVID, https://david.ncifcrf.gov/.ver.6.8) was applied to conduct the enrichment analysis (Dennis et al., 2003). The species was limited to “Homo sapiens”, and the enrichment of pathway was considered significant when the modified fisher exact false discovery rate (FDR) < 0.01. The results of the KEGG pathway and enriched GO terms of biological processes (BP), cell composition (CC), and molecular function (MF) were visualized by the R software package (3.5.2).

### Construction of “Ingredient-Target-Pathway” Network

The “ingredient-target-pathway” networks including the potential ingredients-targets network of GEB against NPP and targets-pathways network of GEB against NPP were constructed by Cytoscape (Shannon et al., 2003). In the network, nodes represent the final active ingredients and targets, while the connections between the nodes represent the interactions between these biological processes and signaling pathways. Three key topological parameters were used to evaluate the topological coefficients between nodes: “degree" (the number of connections between the molecular and target in the core architecture of the network), “betweenness” (the number of shortest paths of a node to the total number of paths through all nodes), and “closeness” (the inverse of the sum of the shortest paths from a node to other nodes in the network).

### Ingredients-Targets Molecular Docking

Molecular docking was used to predict the interactions between core active ingredients of GEB and proteins selected from the center targets from a molecular perspective. 3D structures of active ingredients in SDF (.sdf) format were selected from the PubChem database (https://pubchem.ncbi.nlm), and the crystal structures of the target proteins were downloaded from the PDB database (https://www.rcsb.org/) with a crystal resolution of less than 2 Å. Molecular docking was performed by importing the crystal structure into the Pymol 2.4.1 Software (https://pymol.org/2/) for dehydration, hydrogenation, and ligand separation. Thereafter, Autodock Vina 1.1.2 software was used to construct a crystal structure docking grid box for each target. Then the molecules with the lowest binding energy for each active compound in the docking conformation were allowed for semi-flexible docking by comparing with the original ligands and intermolecular interactions (hydrophobicity, cation-π, anion-π, π-π stacking, hydrogen bonding, etc.). Box center coordinates and size of the box were determined for evaluating the interaction. The results were analyzed and visualized using Pymol, and the numbers of grid points in the three dimensions used in this study were 40 40 40 0.375.

### Molecular Dynamics Simulation of Ligand Complex

The molecular dynamics simulation study is employed to assess the stability and interaction between the protein and ligands after docking. The simulation run was performed for 100 ns using the NVIDIA RTX 1060 GPU accelerated GROMACS 2020 software molecular dynamics package. In the preliminary stage, the Charmm36 force field was used for the protein parameters. The CGenFF server was used for the ligand topology, and a TIP3P water model with appropriate Na^+^/Cl^−^ ions was subsequently generated and neutralized the charge of the system. The system converged to a minimum energy level using the steepest descent method of 50,000 steps and <10.0 kJ/mol force. Then, the equilibration process was conducted with 100 ps for constant NVT (number, volume, and temperature) heating to 300 K, followed by 100 ps for constant NPT (number of particles, pressure, and temperature) with a time step of 2 fs. The bonds of atoms were restrained by recruiting the LINCS algorithm. After the processes of energy minimization and equilibration, the molecular dynamics simulation was conducted the leap-frog algorithm for 100 ns with a time step of 2 fs. The geometrical parameters of the systems, such as root mean square deviation (RMSD) and root mean square fluctuation (RMSF), were determined and compared with the primitive ligand complex.

### Experimental Verification in Chronic Constriction Injury (CCI) Rat Model

Koumine (99% purity) was isolated from GEB as described by Su et al. (Su et al., 2011), and it was dissolved or diluted in sterile physiological saline (0.9% *w/v* NaCl).

Male Sprague–Dawley rats (180–200 g) were purchased from Shanghai Laboratory Animal Center, Chinese Academy of Sciences. The rats were adapted in the condition of 25 ± 2°C with a 12-h light/dark cycle (lights on at 8:00 am) and free access to standard laboratory food and water. The experiments met the requirements of guidelines for animal care and the use of Fujian Medical University. The experimental protocols were reviewed and approved by the Committee of Ethics of the Fujian Medical University (Fujian, China). Animals were assigned into different groups: the sham control group (rats underwent the surgical procedures without any manipulation related to nerve injury), the CCI model group (rats received the vehicle, 0.28, 1.4, 7.0 mg/kg of koumine). The dose used in the experimental assay was based on the published literature (Jin et al., 2018b), and no adverse effects and sedative effects were observed in the rats. The CCI rat model was performed according to the method described by Bennett et al. (Bennett and Xie, 1988).

Behavior tests consist of thermal hyperalgesia and mechanical allodynia tests. The thermal hyperalgesia test using a commercial thermal paw stimulator (PL-200, Chengdu Technology and Market Co., Ltd., Sichuan, China) was evaluated before operation (baseline), drug administration (pre-dosing), and 6, 8, 10, 12, and 14-days after drug administration (post-dosing), and paw thermal withdrawal latency (TWL) was calculated as described by Hargreaves et al. (Hargreaves et al., 1988). The mechanical allodynia test was measured with a commercially available electronic von Frey apparatus (Model 2390; IITC Life Science Inc., Woodland Hills, CA), and each hind paw and mechanical withdrawal latency (MWL) was calculated 30 min after the TWL measurement according to the published literature (Mitrirattanakul et al., 2006). The observer measuring the behaviors was blind to drug pretreatments in all behavioral tests.

Then, rats were anesthetized by chloral hydrate, and the lumber segments (L5-L6) of the spinal cord were dissected, weighed, and stored at −80°C. Then, the lumber segments were homogenized for 30 min in an ice bath with RIPA lysis buffer (CoWin Biosciences, China) containing phosphatase inhibitor (CoWin Biosciences, China) after ultrasonic crushing. Protein concentrations were determined using an enhanced BCA protein assay kit (Beyotime Biotech Inc., China), and the protein samples were stored at −80°C until use. Total protein samples were separated by sodium dodecyl sulfate polyacrylamide gel electrophoresis and transferred onto the nitrocellulose membrane. The membrane was blocked with 5% non-fat dried milk in tris buffer for 1 h at room temperature and washed with tris-buffered saline and tween 20 every 10 minutes for three times. Then, the membrane was incubated with antibodies (EGFR rabbit pAb: A11577, 1:500, ABclonal; JAK1 rabbit pAb: A5534, 1:500, ABclonal; AKT1 rabbit mAb: A17909, 1:500, ABclonal; β-actin rabbit mAb: AF1186, 1:1,000, Beyotime) overnight at 4°C. After incubation with the appropriate secondary antibodies (HRP-labeled goat anti-rabbit IgG: A0208, 1:1,000, Beyotime) at room temperature for 1 h, the protein blots were visualized in the ChemiDoc XRS imaging system (Bio-Rad, CA).

## Results

### Putative Targets of GEB Against NPP

**FIGURE 2:**
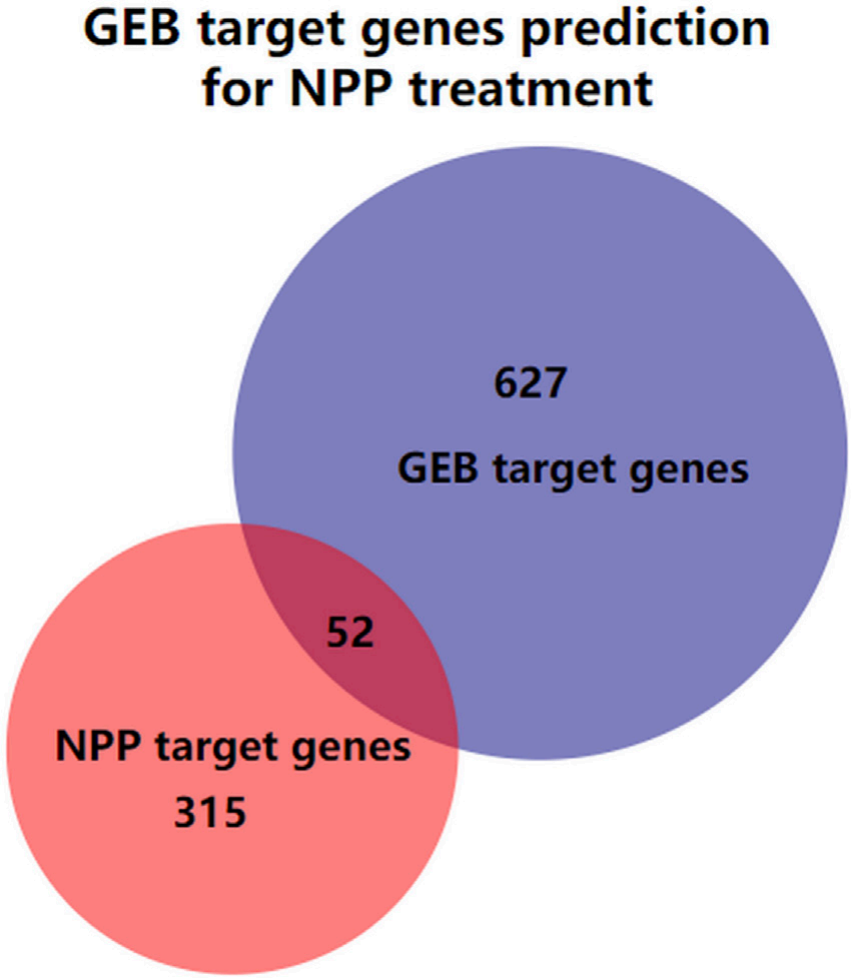
The Venn diagram illustrating the intersection of the GEB target genes and NPP target genes. The blue circle is the GEB target genes, the red circle is the NPP target genes, and the overlap of the two circles indicates GEB target genes prediction for NPP treatment.

A total of 98 compounds in GEB were retrieved from published literature and online databases, and 57 potentially active ingredients were filtered by OB and DL provided by SwissADME (Table 1). 679 potential targets were eventually predicted based on the SwissTargetPrediction after eliminating duplicate targets (Supplementary Table S1). 1,047 targets related to NPP were obtained through the Gene Cards database. Out of these targets, 367 potential targets were finally screened out with a disease relevance score ≥3 (Supplementary Table S2). Subsequently, as shown in Figure 2, 679 GEB ingredients-related targets were intersected with 367 NPP disease-related target genes using Venn diagrams to identify 52 putative targets between GEB and NPP, which were considered candidate targets of GEB against NPP.

**TABLE 1:** Information of the active compounds in GEB for network analysis.

NO.	Name	Compound CID	MW	MF	Source
1	N-methoxyanhydrovobasinediol	102004539	338.4	C_21_H_26_N_2_O_2_	References
2	Humantenirine	11,132,403	370.4	C_21_H_26_N_2_O_4_	TCMID, TCMSP, Reference
3	N-desmethoxyrankinidine	5316594	310.4	C_19_H_22_N_2_O_2_	TCMID, TCMSP, References
4	Humantendine	5490912	342.4	C_19_H_22_N_2_O_4_	References
5	11-Methoxygelsemamide	5319437	355.4	C_21_H_25_NO_4_	TCM-Taiwan, References
6	Gelsevirine	14217344	352.4	C_21_H_24_N_2_O_3_	TCMID, BATMAN-TCM, TCM-Taiwan, References
7	Gelsenicine	21123652	326.4	C_19_H_22_N_2_O_3_	References
8	19-Oxogelsenicine	102185549	398.4	C_21_H_22_N_2_O_6_	References
9	Gelsedine	21589070	328.4	C_19_H_24_N_2_O_3_	TCMID, BATMAN-TCM, TCM-Taiwan, References
10	Gelsemamide	5317542	340.4	C_20_H_24_N_2_O_3_	TCMID, TCMSP, References
11	19-Z-akuammidine	44583830	352.4	C_21_H_24_N_2_O_3_	References
12	Dihydrokoumine	5316727	308.4	C_20_H_24_N_2_O	BATMAN-TCM, References
13	(19R)-kouminol	NA	324.2	C_20_H_24_N_2_O_2_	References
14	(19S)-kouminol	NA	324.2	C_20_H_24_N_2_O_2_	References
15	19-(R)-hydroxydihydrokoumine	50278496	324.4	C_20_H_24_N_2_O_2_	TCMID, BATMAN-TCM, TCM-Taiwan, References
16	19-(S)-hydroxydihydrokoumine	5318193	324.4	C_20_H_24_N_2_O_2_	References
17	20-hydroxydihydrorankinidine	101606432	358.4	C_20_H_26_N_2_O_4_	References
18	N-desmethoxyhumantenine	5316593	324.4	C_20_H_24_N_2_O_2_	References
19	15-hydroxyhumantenine	101606434	370.4	C_21_H_26_N_2_O_4_	TCMID, TCMSP, References
20	Rankinidine	6439112	340.4	C_20_H_24_N_2_O_3_	TCMID, TCMSP, References
21	Humantenmine	158212	326.4	C_19_H_22_N_2_O_3_	TCMID, BATMAN-TCM, TCM-Taiwan, Reference
22	11-Hydroxyrankinidine	5318332	356.4	C_20_H_24_N_2_O_4_	TCMID, References Lin et al. (2021)
23	11-Hydroxyhumantenine	5318224	370.4	C_21_H_26_N_2_O_4_	TCMID, References Lin et al. (2021)
24	11-methoxyhumantenine	44583832	384.5	C_22_H_28_N_2_O_4_	TCMID, TCMSP, BATMAN-TCM, References Lin et al. (2021)
25	19R-hydroxydihydrogelsevirine	5318192	370.4	C_21_H_26_N_2_O_4_	References Lin et al. (2021)
26	19S-hydroxydihydrogelsevirine	5318192	370.4	C_21_H_26_N_2_O_4_	References Lin et al. (2021)
27	Gelseoxazolidinine	102297300	428.5	C_23_H_28_N_2_O_6_	References Lin et al. (2021)
28	Gelsevanillidine	136811988	460	C_27_H_28_N_2_O_5_	References Lin et al. (2021)
29	Gelselegine	10948335	358.4	C_20_H_26_N_2_O_4_	TCMID, References Lin et al. (2021)
30	11-Methoxy-19-R-hydroxygelselegine	5319453	404.5	C_21_H_28_N_2_O_6_	References Lin et al. (2021)
31	19α-hydroxygelsamydine	102003053	524.6	C_29_H_36_N_2_O_7_	References Lin et al. (2021)
32	gelsamydine	5317540	508.6	C_29_H_36_N_2_O_6_	TCMID, References Lin et al. (2021)
33	gelegamine E	101467881	370.4	C_20_H_22_N_2_O_5_	References Lin et al. (2021)
34	gelegamine C	101467879	514.4	C_21_H_27_IN_2_O_5_	References Lin et al. (2021)
35	Gelegamine A	101467877	384.4	C_21_H_24_N_2_O_5_	References Lin et al. (2021)
36	Gelegamine B	101467878	384.4	C_21_H_24_N_2_O_5_	References Lin et al. (2021)
37	19Z- 16- epi- voacarpine	NA	368.2	C_21_H_24_N_2_O_4_	References Lin et al. (2021)
38	11-Methoxyhumantenmine	NA	356.2	C_20_H_24_N_2_O_4_	References Lin et al. (2021)
39	GELSENINE	NA	358.19	C_20_H_26_N_2_O_4_	References Lin et al. (2021)
40	21- Oxokoumine	NA	320.1	C_20_H_20_N_2_O_2_	References Lin et al. (2021)
41	Furanokoumine	NA	322.1	C_20_H_22_N_2_O_2_	References (Lin et al., 2021)
42	Koumidine	44584550	294.4	C_19_H_22_N_2_O	TCMID, TCMSP, BATMAN-TCM, References Lin et al. (2021)
43	Gelebolines A	NA	320.15	C_20_H_20_N_2_O_2_	References Lin et al. (2021)
44	Gelebolines B	NA	334.2	C_21_H_22_N_2_O_2_	References Lin et al. (2021)
45	Gelebolines C	NA	334.1	C_20_H_18_N_2_O_3_	References Lin et al. (2021)
46	19E- 16- epi- voacarpine	NA	368.2	C_21_H_24_N_2_O_4_	References Lin et al. (2021)
47	19- Z- taberpsychine	5321582	310.4	C_20_H_26_N_2_O	References Lin et al. (2021)
48	Koumicine N- oxide	NA	322.4	C_20_H_22_N_2_O_2_	References Lin et al. (2021)
49	Nb-methylgelsedilam	NA	328.14	C_18_H_20_N_2_O_4_	References Lin et al. (2021)
50	15-hydroxy-Nb-methylgelsedilam	NA	344.1	C_18_H_20_N_2_O_5_	References Lin et al. (2021)
51	Gelsesyringalidine	136704418	490.5	C_28_H_30_N_2_O_6_	References Lin et al. (2021)
52	14-Dehydroxygelsefuranidine	1,02417029	404.5	C_24_H_24_N_2_O_4_	References Lin et al. (2021)
53	Humantenoxenine	NA	368.17	C_21_H_24_N_2_O_4_	References Lin et al. (2021)
54	15-Hydroxyhumantenoxenine	101606434	370.4	C_21_H_26_N_2_O_4_	TCMID
55	Kounaminal	102260292	363.5	C_22_H_25_N_3_O_2_	References Lin et al. (2021)
56	Dehydrokoumidine	119077162	292.4	C_19_H_20_N_2_O	References Lin et al. (2021)
57	Koumine	91895267	306.4	C_20_H_22_N_2_O	TCMID, TCMSP, BATMAN-TCM, TCM-Taiwan, References Lin et al. (2021)

NA, not applicable.

### PPI Network of Targets for GEB Against NPP

**FIGURE 3:**
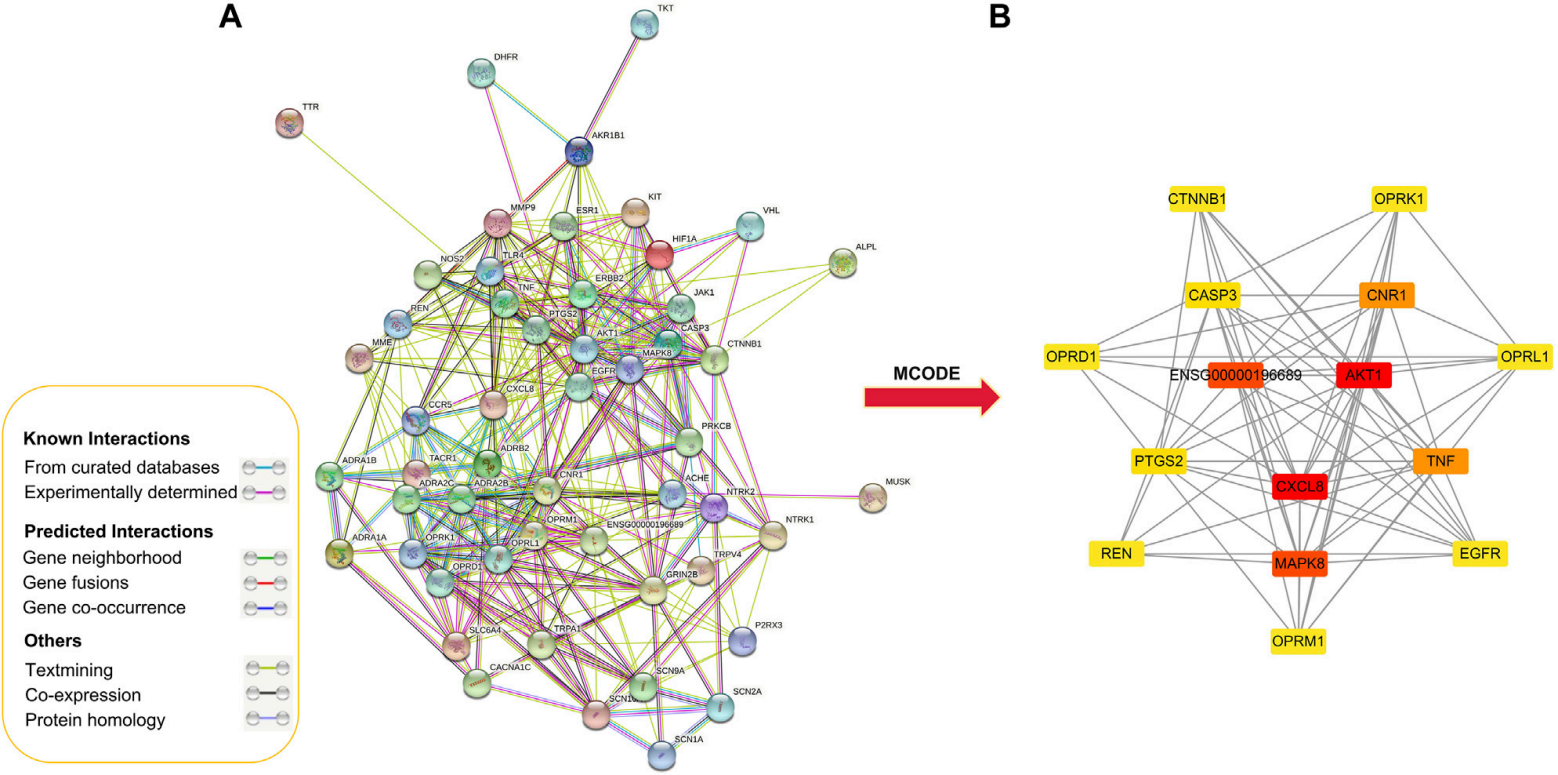
Protein-protein interaction network for GEB in treatment for NPP. **(A)** Protein-protein interactions among the 52 core targets. Network nodes represent proteins, and edges represent protein-protein associations. **(B)** PPI network of the hub genes using MCODE plugin. The color from red/orange/yellow indicated the different importance of the nodes in the whole PPI network. That is, the redder the retangle is, the more important the node is in the PPI network.

PPI analysis was performed on 52 putative common targets with String database as illustrated in Figure 3. The numbers of edges were corresponding to the strength of correlation between two target proteins. The top 15 proteins in the center of the PPI network (Table 2), namely AKT1, ENSG00000196689, TNF, CASP3, CXCL8, MAPK8, OPRM1, EGFR, OPRL1, CNR1, PTGS2, CTNNB1, REN, OPRD1, and OPRK1. These proteins were speculated as the core target to play a significant role in the treatment of NPP.

**TABLE 2:** Key targets of the PPI network.

NO.	Common name	Degree
1	AKT1	29
2	ENSG00000196689	27
3	TNF	25
4	CASP3	25
5	CXCL8	24
6	MAPK8	24
7	OPRM1	23
8	EGFR	23
9	OPRL1	20
10	CNR1	20
11	PTGS2	18
12	CTNNB1	18
13	REN	16
14	OPRD1	16
15	OPRK1	16

### GO Function and KEGG Pathway Enrichment Analysis

**FIGURE 4:**
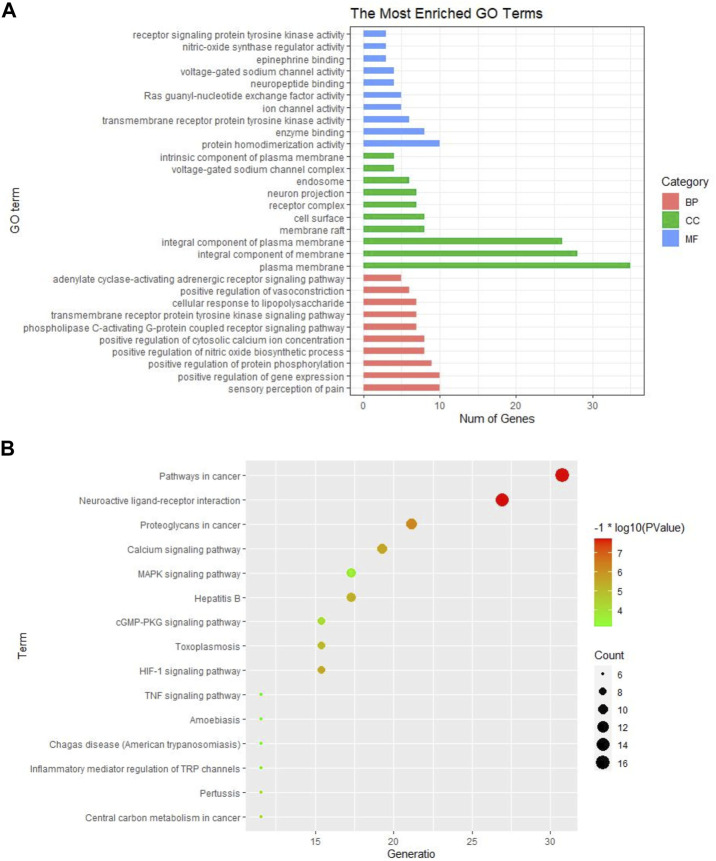
GO and KEGG pathway enrichment analysis of GEB in treatment for NPP. **(A)** GO analysis of significant items of 52 common targets in different functional groups (BPs, CCs, MFs) **(B)** The top 15 KEGG pathways based on their *p*-values. The larger the circle, the greater the number of the target genes in the term. Also, color highlights the size of the FDR: the redder the color, the more significant the value.

The GO and KEGG pathway analysis were performed on the 52 common targets to further explore the possible mechanisms of GEB against NPP. A total of 184 BPs, 34 CCs, and 35 MFs were obtained in GO analysis (Supplementary Table S3). The top 10 terms in BPs, MFs, and CCs were shown in Figure 4A. It was suggested that GEB attenuated NPP may be through the sensory perception of pain, positive regulation of gene expression/protein phosphorylation/nitric oxide biosynthetic/cytosolic calcium ion concentration, and phospholipase C-activating G-protein coupled receptor signaling pathway. Meanwhile, the target protein was mainly distributed in the plasma membrane and involved in protein homodimer activity, enzyme binding, transmembrane receptor protein tyrosine kinase activity, etc.

KEGG pathway analysis identified 73 pathways, including neuroactive ligand-receptor interactions, calcium signaling pathway, MAPK signaling pathway, HIF-1 signaling pathway, cGMP-PKG signaling pathway, Rap1 signaling pathway, inflammatory mediators regulating TRP channels, and TNF signaling pathway (Supplementary Table S4). The top 15 KEGG pathways based on their *p*-values were selected to generate a bubble chart for visualization (Figure 4B).

### “Ingredient-Target-Pathway” Network Construction

**FIGURE 5:**
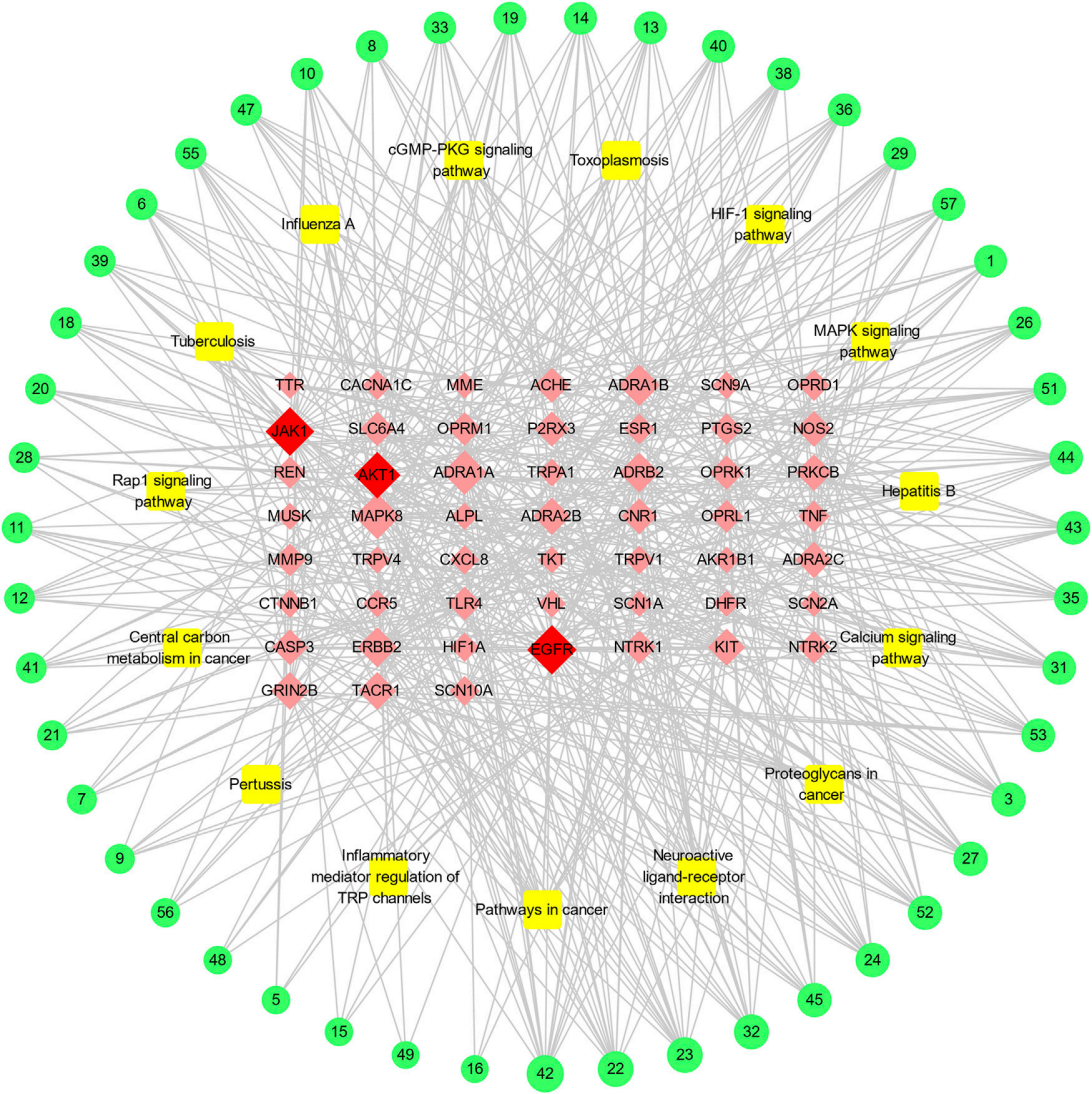
The “ingredient-target-pathway” network for GEB in treatment for NPP. The outer green circle represents active ingredient in GEB, the yellow square indicates the potential pathways, and the red diamond stands for the potential target. For corresponding active ingredient names, refer to Supplementary Table1. the potential target genes shared by GEB and NPP, the orange triangle stands for the active ingredient in GEB, and the green diamond indicates the potential pathways. The size of the node represents the degree values.

Based on the active ingredients related to the target genes, the active ingredients–disease targets–pathway network with 114 nodes and 556 edges was constructed (Figure 5). Of the 99 nodes, 47 active ingredients nodes, 52 target nodes, and 15 pathways were involved. The top three active ingredient nodes with the most edges were 11-hydroxyrankinidine, 11-hydroxyhumantenine, and gelseoxazolidinine. The average values of the degree values, node betweenness, and closeness of the three topological features of these active ingredients were 13.8, 0.0402, and 0.4424, respectively. The top three target nodes with the most degrees were EGFR, JAK1, and AKT1. The average values of the degree values, node betweenness, and closeness of the three topological features are 23, 0.0855, and 0.4658, respectively.

### Ingredients-Targets Molecular Docking

The molecular docking technology was used to further evaluate the interaction between the ingredients and the targets, and verify the accuracy of the network analysis. The binding strength of the ligand and the receptor depends on the change in the binding energy. The lower the binding energy between the ligand and the receptor, the more stable and greater the possibility of interaction of ligand-receptor binding. The top three core active ingredients and three targets were used as receptors and ligands, respectively. In addition, koumine was also acted as a receptor for target docking simulation since it was one of the most studied ingredients of GEB (Table 3). The molecular docking results demonstrated that each ingredient could match well with each target to verify the accuracy of the prediction network construction. 11-Hydroxyrankinidine had the highest affinity for the target of EGFR (PDB: 5HG7), AKT1 (PDB: 3L9M), and JAK1 (PDB: 4E5W), and had the lowest binding energy with EGFR, which indicated that 11-hydroxyrankinidine had a strong affinity with the active pocket of EGFR.

**TABLE 3:** Virtual molecular docking of active ingredients of GEB and targets.

Name	The number of hydrogen bond	Amino acid residue	Target	Binding Energy/kcal·mol^−1^
11-Hydroxyrankinidine	3	MET-793 (2.8)	EGFR	−8.7
		CYS-797 (3.3)		
	3	ARG-1007 (2.7)	JAK1	−6.5
		LEU-959 (2.8)		
		LEU-959 (2.5)		
	1	GLU-170 (2.8)	AKT1	−8
11-Hydroxyhumantenine	1	CYS-797	EGFR	−7.6
	1	GLU-883	JAK1	−5.9
	NA	NA	AKT1	−6.4
Gelseoxazolidinine	1	SER-720	EGFR	−6.4
	NA	NA	JAK1	−5.8
	1	ASN-20	AKT1	−2.5
Koumine	1	CYS-797 (3.2)	EGFR	−7.2
	NA	NA	JAK1	−6.2
	1	ASP-184 (3.1)	AKT1	−7.4

NA, not applicable.

**FIGURE 6:**
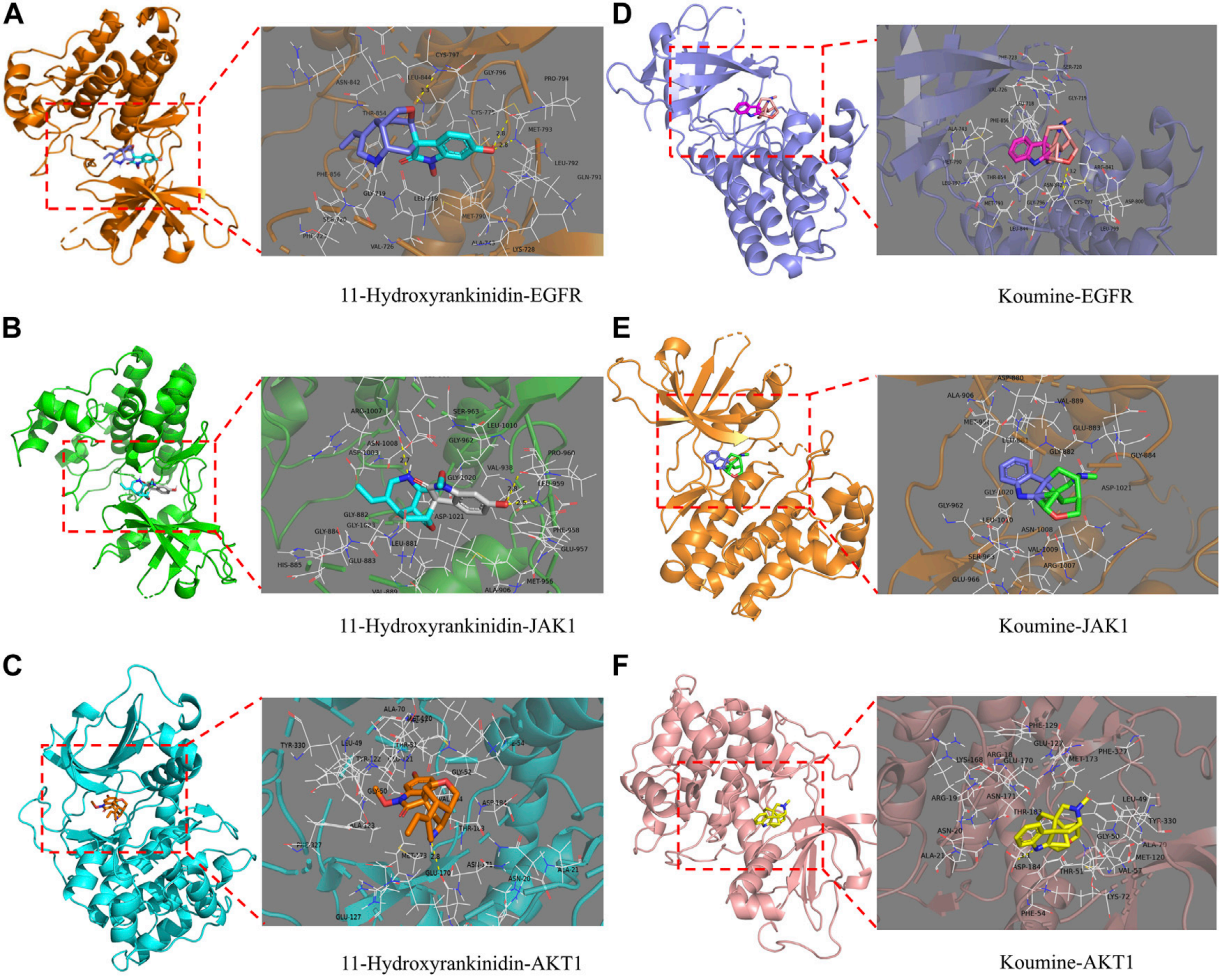
Virtual docking of the binding of EGFR, JAK1, and AKT1 with 11-Hydroxyrankinidine **(A–C)** and koumine **(D–F)** shown as 3D diagrams.

Multi-conformation molecular docking results were visualized using Pymol software in Figure 5. The 11-hydroxyrankinidine-EGFR complex was stabilized by three hydrogen bonds with potential residues including MET-793 (2.8 Å) and CYS-797 (3.3 Å) (Figure 6A). The 11-hydroxyrankinidine- JAK1 complex was mainly through three hydrogen bonding interactions of ARG-1007 (2.7 Å), LEU-959 (2.8 Å), and LEU-959 (2.5 Å) (Figure 6B). The 11-hydroxyrankinidine- AKT1 complex interacted with the residue GLU-170 (2.8 Å) through one hydrogen bond (Figure 6C). The results of virtual docking between koumine and the targets EGFR, JAK1, and AKT1 were denoted in Figure 6D–F respectively.

### Molecular Dynamics Simulation of Ligand Complex

**FIGURE 7:**
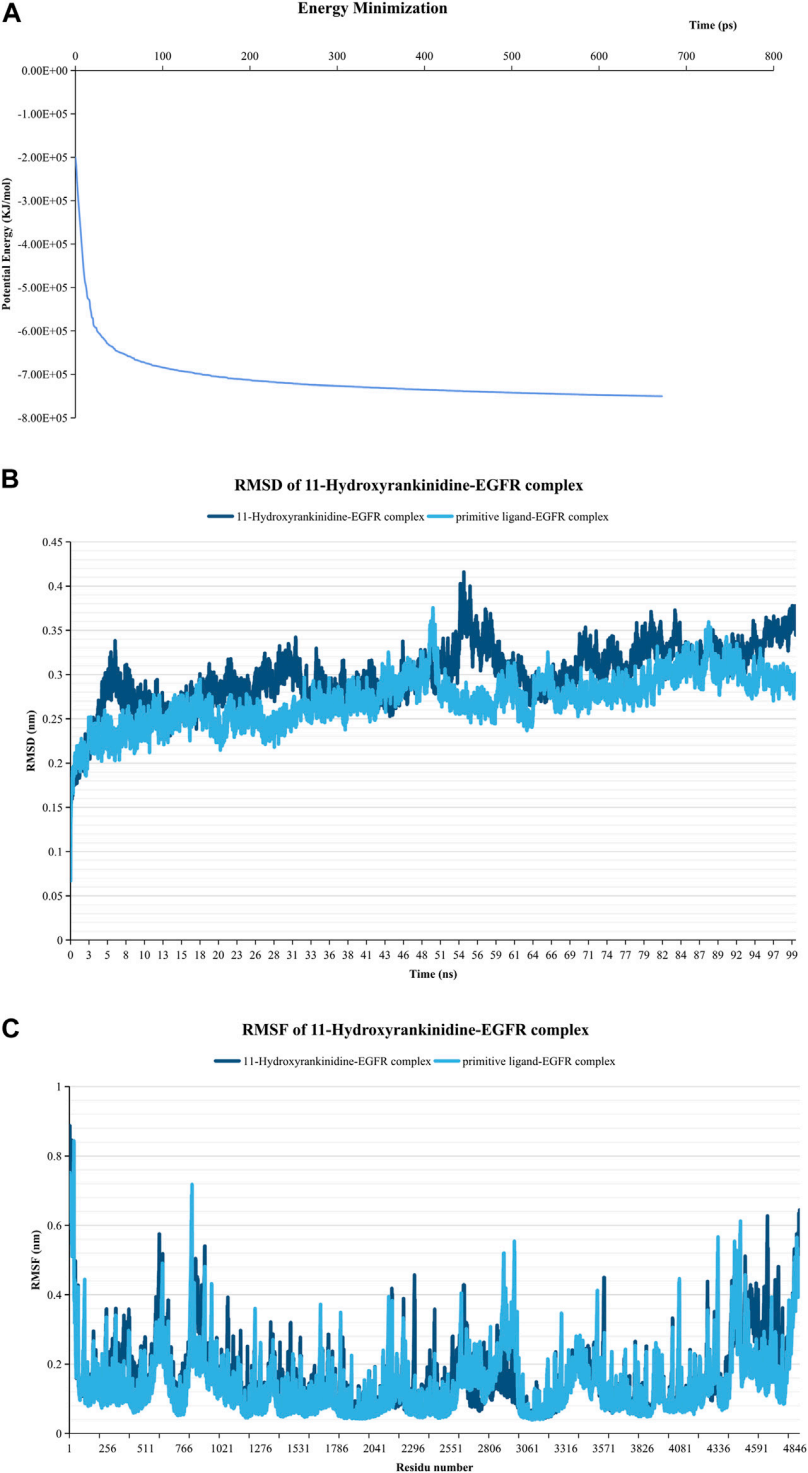
Molecular dynamics simulation of the 11-Hydroxyrankinidine-EGFR complex. **(A)** Energy minimization for molecular dynamics simulation. **(B)** RMSD profiles of complex conducted during 100 ns. **(C)** RMSF profiles of the complex. The primitive ligand of EGFR is ibrutinib.

Since 11-Hydroxyrankinidine has the lowest binding energy with EGFR, 11-hydroxyrankinidin-EGFR complex was selected for molecular dynamics simulation to elaborate the dynamic interactions between the protein-ligand complex and confirm the molecular docking results. The system’s potential energy converged within 100 ps (Figure 7A), which indicated that the simulation system was well prepared. The RMSD graph shown in Figure 7B demonstrated that a consistently stable structure of 11-hydroxyrankinidine-EGFR complex throughout the simulation period. The mean (±SD) RMSD of the 11-hydroxyrankinidine-EGFR complex (0.254 ± 0.018 nm) was similar with primitive ligand complex (0.263 ± 0.026 nm), even lower than that of the reference ligand after 26 ns. Meanwhile, the RMSF shows comparatively restricted fluctuation in the protein residues (Figure 7C). Mean (±SD) RMSF values of 11-hydroxyrankinidine-EGFR complex and primitive ligand complex were 0.147 ± 0.097 nm and 0.141 ± 0.095 nm, respectively. There were no apparent differences for all complexes in the ligand RMSF. This observation demonstrated that the molecules were capable of forming stable interactions with the protein during simulation.

### Anti-NPP Effects of Koumine in CCI Rat Model

**FIGURE 8:**
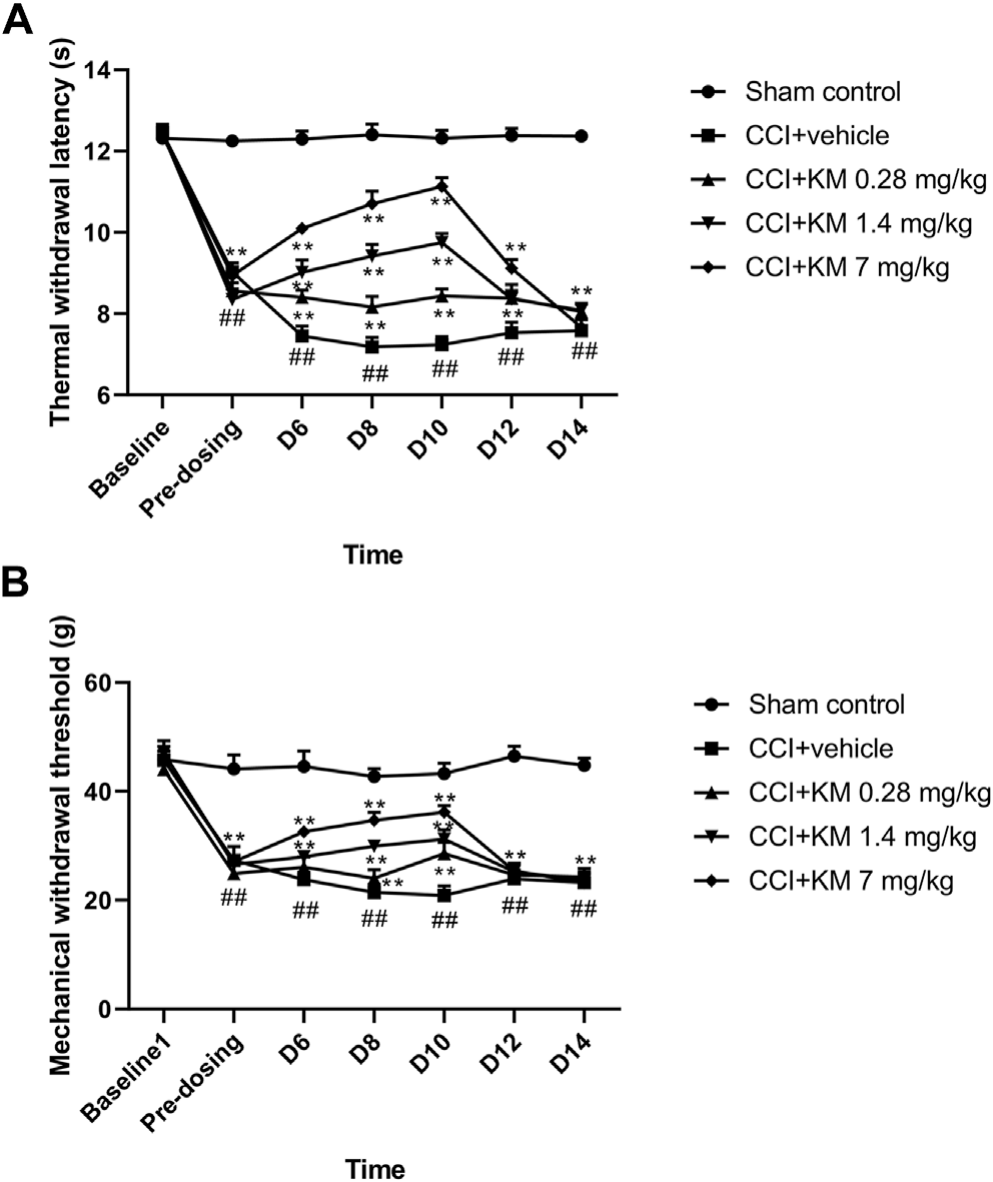
The anti-NPP effect of koumine in sciatic nerve chronic constriction injury rats. **(A)** The time course of the effect of koumine on the thermal withdrawal latency. **(B)** The time course of the effect of koumine on the mechanical withdrawal threshold. The data are presented as the mean ± SD (n = 6) and were analyzed using two-way repeated analysis of variance (ANOVA) followed by Bonferroni’s post hoc test, ^##^ *p* < 0.01 compared with the sham control group; ^**^ *p* < 0.01 compared with the vehicle control group. KM, koumine.

In the current study, the TWL to thermal stimulation and the MWL to mechanical stimulation of the CCI rats were significantly decreased compared with the sham group (*p* < 0.001). Koumine attenuated the CCI-induced NPP effect in the dose-dependent and time-dependent manner (Figure 8). The results indicated that the maximum anti-NPP effect was reached on day 10 and administration of koumine (7 mg/kg) exhibited the maximum pharmacological effect to reverse NPP.

### Validation of Predicted Target Protein With Western Blotting

**FIGURE 9:**
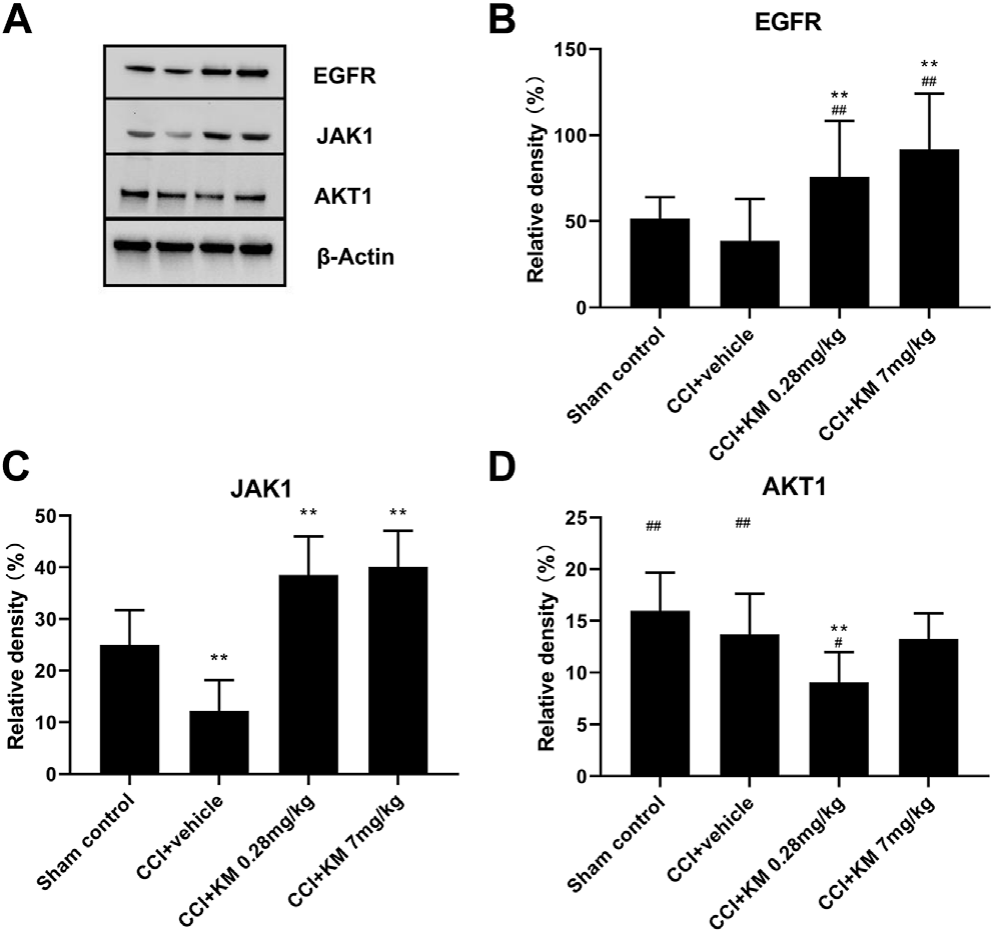
Western blot **(A)** and relative protein expression of EGFR **(B)**, JAK1 **(C)**, and AKT1 **(D)**. The data are presented as the mean ± SD (n = 6) and were analyzed using two-way repeated analysis of variance (ANOVA) followed by Bonferroni’s post hoc test, ^##^ *p* < 0.01 compared with the sham control group; ^**^ *p* < 0.01 compared with the vehicle control group. KM, koumine.

As shown in Figure 9, the relative protein expression of EGFR in koumine treated group was higher than that in sham control and CCI + vehicle groups, which indicated that koumine reversed the CCI-induced downregulation of EGFR in a dose-dependent manner. Similarly, the activation of JAK1 was observed in koumine treated group. In addition, the protein expression of AKT1 in the low-dose group of koumine was lower than that in the sham control group. However, no significance was observed between the sham control group and the low-dose group of koumine. The reason for this may be related to the complex interaction of pathways adjusted by multiple ingredients, which needs to study further.

## Discussion

To the best of our knowledge, this is the first systematical study for exploring the potential pharmacological and molecular mechanisms of GEB against NPP from the network pharmacology and experimental perspective. The major findings were as follows: (Vos et al., 2012): a total of 52 proteins were considered as potential targets associated with NPP according to the “ingredient-target-pathway” network, and the top four targets were EGFR, JAK1, AKT1, and MAPK8; (Andrew et al., 2014); GO and KEGG enrichment analysis revealed that GEB was involved in phosphorylation reactions and nitric oxide synthesis processes. It also participated in 73 pathways in the pathogenesis of NPP, including the neuroactive ligand-receptor interaction signaling pathway, calcium signaling pathway, and MAPK signaling pathway; (van Hecke et al., 2014); a total of 47 active alkaloids might play a synergistic role in the treatment of NPP. Among them, 11-hydroxyrankinidin matched the active pockets of EGFR, JAK1, and AKT1 proteins, with the strongest affinity, suggesting that it may be an essential component of GEB in the treatment of NPP; (Jensen et al., 2011); koumine reversed the CCI-induced downregulation of EGFR and JAK1. These findings revealed the complex network relationship of GEB in the “multi-ingredient, multi-target, multi-pathway” mode, and explained the synergistic regulatory effect of each complex ingredient of GEB based on the holistic view of TCM.

By predicting targets and constructing the PPI network for the common targets of GEB and NPP, 52 targets were distinguished to investigate the possible mechanism of GEB against NPP. The top targets, EGFR, JAK1, AKT1, and MAPK8, were thought to be involved in NPP regulation. The first target, EGFR is expressed on peripheral nerves, and it belongs to the ErbB family of receptor tyrosine kinases (Neto et al., 2017). After nerve injury, EGFR is upregulated in the nervous system and has been proposed as a target for the treatment of NPP (Liu et al., 2006). EGFR also plays a key role in many intracellular signaling pathways, such as phosphatidylinositol 3-kinase, MAPK, and ErbB signaling pathways (Tao et al., 2013; Borges et al., 2021), which is coherent with the results of KEGG and molecular docking in the present study. Furthermore, treatment of NPP with EGFR-Inhibitors (EGFR-Is) significantly relieved the pain of the majority of patients, which has been reported in clinical and preclinical studies (Kersten et al., 2015; Kersten et al., 2019). The second target, JAK1 is the core member of the JAK family and stimulates the phosphorylation of STAT3 through particular domains (Wang et al., 2020b). The JAK/STAT signaling pathway directly or indirectly affects the action, expression, and regulation of a multitude of cytokines in mediating various mechanisms underlying pain (Simon et al., 2021). JAK1 was increased in spinal nerve ligation triggered NPP rat models (Wang et al., 2020b), which was validated to be a potential therapeutic target of NPP. A previous study revealed that dexmedetomidine had the potential to alleviate NPP by regulating the JAK/STAT pathway in chronic constriction injury rats (Xun and Zheng, 2020). It is suggested that the active ingredients may exert therapeutic effects via regulating JAK1 expression or its function. Another target of AKT, a key downstream substrate in the PI3K pathway, is associated with diverse biological processes (Sun et al., 2006). The phosphorylation of the PI3K/AKT pathway in the spinal cord contributes to the activation of the transcription factor nuclear factor κB and the release of the inflammatory mediators and finally leads to the development of NPP (Chen et al., 2017). Therefore, the PI3K/AKT pathway is likely a novel target for GEB against NPP. As the potential downstream pathways of the EGFR, accumulating evidence showed that the MAPK family contributes to the regulation of pain hypersensitivity in different injury conditions via phosphorylation activation (Gao et al., 2009; Ji et al., 2009). A slow (>3 days) and persistent (>21 days) activation of MAPK8, known as c-Jun N-terminal kinase 1 (JNK1), could be induced in the spinal nerve ligation model (Zhuang et al., 2006). Although the current evidence was limited, it was reported that the latent mechanism of GEB may be related to the activation of MAPK *in vitro* and *vivo* studies (Yuan et al., 2016; Huang et al., 2021). These pieces of evidence exemplify that the pharmacological activity of GEB against NPP is due to the interaction of these key targets. Moreover, multi-target therapeutics approaches for GEB in control of NPP form a basis for further research on the mechanism of GEB and the development of novel therapeutic approaches in the future.

The related pathways and biological processes of GEB in the treatment of NPP also reflected the multi-pathway characteristics of TCM. Based on GO functional enrichment analysis, the biological process mainly focused on the different phosphorylation and nitric oxide (NO) synthesis processes, which indicated that a potentially novel mechanism for pharmacological intervention of GEB against NPP. Protein phosphorylation plays a key role in the cellular regulatory mechanism of enzymes and receptors. Most of the above-mentioned target activation is involved in the phosphorylation process. For example, phosphorylation of AKT at Thr308 and Ser473 mediate pain behavior through the PI3K/AKT signal pathway (Sun et al., 2006). Notably, GEB reduced the oxidative stress and inflammatory reaction in a phosphorylation state-dependent modulation manner (Yuan et al., 2019; Luo et al., 2020; Wu et al., 2020). In addition, NO is an important neurotransmitter and modulates a wide variety of physiological functions. It has been illustrated that NO mediates the analgesic effect of opioids and other analgesic substances through activation of the cGMP–PKG–ATP-sensitive K^+^ channels pathway (Cury et al., 2011), which is consistent with the results of KEGG enrichment. It was also reported that koumine decreases the productions of NO and pro-inflammatory mediators in RAW264.7 cells (Yuan et al., 2016). These findings may partially support our prediction on GO and KEGG pathway enrichment analysis. Furthermore, the KEGG pathway enrichment analysis showed that GEB may participate in neuroactive ligand-receptor interaction, calcium, and inflammatory mediator regulation signaling pathways. Neuroactive ligand-receptor interaction refers to the stereoselectivity between neuroactive steroids and receptors (Wang et al., 2017). Neuroactive steroids act as regulators to influence the modulation of neuronal activity (Smith, 1994). As confirmed by existing literature, the neurosteroid allopregnanolone exert a positive allosteric regulation of the GABA receptor and was activated by GEB and its active alkaloids (Zhang and Wang, 2015). Mirtazapine affects neuroactive steroid composition similarly to koumine with an enhanced formation of 3α-hydroxysteroid dehydrogenase neuroactive steroids (Schüle et al., 2006; Qiu et al., 2015). Based on these, we infer that GEB might serve a crucial role in NPP by influencing the pathway of neuroactive ligand-receptor interaction pathway. In addition, increased expression of voltage-gated calcium channels at dorsal root ganglia and presynaptic terminals increases the excitability of nerve and lead to NPP (Cohen and Mao, 2014).

Inflammatory mediator regulation is another interesting pathway described by KEGG. An increasing number of studies have indicated that inflammatory responses play a key role in the development of NPP (Watkins et al., 2003). Cytokines, which are essential for the induction and maintenance of pain (Zhang and An, 2007), are primarily secreted by immune cells. Pro-inflammatory cytokines (e.g., TNF-α, IL-1β, IL-6, and IL-17) are evoked in inflammatory responses after nerve injury through intracellular mediators, while anti-inflammatory (IL-4, IL-10, TGF-β) signaling molecules show analgesic properties (Hung et al., 2017). The PI3K/AKT signaling pathway is an inflammatory pathway that may be mediated by TNF-α in osteoarthritis, and TNF-α inhibitor treatment significantly reduced the expression of IL-1, IL17a, and IL8 in synovial fibroblasts (Li et al., 2018). In addition, the TNF-induced cutaneous hypersensitivity to mechanical or thermal stimulation is also associated with the cAMP-dependent protein kinase (PKA) pathway and the p38 mitogen-activated protein kinase (MAPK) pathway (Zhang et al., 2002; Schäfers et al., 2003), which are all presented in our network results and provides a systematic and macro perspective to understand their interactions. Another cytokine, IL-17, has shown the potential effect on allodynia. Exogenous IL-17 administration may increase the activity of the transient receptor protein vanilloid 4 (TRPV4) (Hung et al., 2017), which was discovered in network pharmacology. Furthermore, gelsemine and koumine have been shown to inhibit the overexpression of pro-inflammatory cytokines in mice and rats (Jin et al., 2018b; Chen et al., 2020). These above evidenced improve our confidence that GEB can restore the imbalance between pro-inflammatory cytokines and anti-inflammatory cytokines and thus promote its antinociceptive effects.

Based on the “ingredient-target-pathway” network, 47 pivotal active ingredients related to NPP were obtained, such as 11-hydroxyrankinidine, 11-hydroxyhumantenine, gelsamydine, koumidine, and gelebolines C. These ingredients might play a synergistic role in the treatment of NPP. Structurally, the 47 phytochemicals have exhibited diversity. Molecular docking results have disclosed that 11-hydroxyrankinidine, 11-hydroxyhumantenine, gelsamydine, and koumine could bind autonomously with the active pocket of EGFR, JAK1, and AKT1 to form a complex with a relatively stable structure through hydrogen bonds and other interactions. Among them, 11-hydroxyrankinidine has the lowest binding energy and the highest affinity to EGFR. Encouragingly, it was observed that the RSMD and RSMF profiles of the 11-hydroxyrankinidine-EGFR complex were relatively stable, which indicated 11-hydroxyrankinidine showed promising inhibitory activity in NPP. Due to the accessibility, the extracts and monomers derived from GEB have been found to possess anti-NPP biological activity, especially some of the active ingredients, such as koumine (Xu et al., 2012; Qiu et al., 2015; Jin et al., 2018a; Jin et al., 2018b), gelsenicine (Liu et al., 2011), and gelsemine (Zhang et al., 2013; Wu et al., 2015; Chen et al., 2020). To further verify the hypothesis of network pharmacology, that is, the active alkaloids obtained through network pharmacology act on the target to exert an anti-NPP effect, the behavior test and western blotting were applied to evaluate the anti-NPP effects of koumine as well as its molecular mechanism after the analysis of network pharmacology, molecular docking, and molecular dynamics simulation. It was suggested that koumine could upregulate the protein expression of EGFR and JAK1 to achieve the anti-NPP action. Combined with the prediction results, it was reasonable to speculate that koumine or GEB-derived ingredients contributed to the anti-NPP effect through some pain-related targets. Most of the indole alkaloids have a similar skeletal structure. Therefore, it is worthy of further examination for the therapeutic effects of 11-hydroxyrankinidine against NPP both *in vitro* and *in vivo*, though the current data on 11-hydroxyrankinidine is extremely limited. Furthermore, traditional TCM usually is thought to act in synergy to achieve a holistic therapeutic outcome, suggesting the understanding of the synergistic action of these alkaloids with a holistic view.

This present study also has several limitations. Firstly, given the limitations of network pharmacology, the public databases investigated in the study would be constantly updated so some ingredients and targets information has partly lagged. The chemical fingerprint may be a better choice for network construction and mechanism exploration. Secondly, SwissTargetPrediction is a ligand-based tool for predicting the interacting targets of small molecules, which is useful for understanding the molecular mechanisms underlying a specific phenotype or bioactivity, as well as assessing the possibility of repurposing therapeutically-relevant compounds. It is based on the so-called “similarity principle,” which states that two similar molecules are likely to have similar properties. However, when there are few (or no) known active ligands for a target of interest, their predictive reliability suffers. Furthermore, when molecules with high structural similarities but different biological activities for the same target coexist, a limitation may also exist. Thirdly, the validation experiment in the current study only provided limited protein, and more targets and pathways may be studied further, especially the possibility of influence and correlations with cytokines. Besides, although 11-hydroxyrankinidine showed promising inhibitory activity in network pharmacology, it is not the most abundant ingredient in GEB. Due its inaccessibility, there has been no report on its pharmacological activities to date. Therefore, koumine was selected in validation in the experiment as a substitute, based on the fact that it is a high-content active alkaloid of GEB that is easily obtained. Furthermore, molecular docking results demonstrated that koumine bonded to the ligand stably. For further study, we can also use pH-zone refining counter-current chromatography to purify the active monomer (Su et al., 2011), for example, 11-hydroxyrankinidine, to determine its analgesic effect in the future.

In the present study, the active ingredient and anti-NPP mechanism of GEB were mapped using network pharmacology, molecular docking, molecular dynamics simulation, and bioinformatics approach. It could promote the understanding of the synergistic action of GEB with a holistic view to explore the key ingredients, targets, and pathways. Different from the current mode of single-target pharmacology in TCM, network pharmacology based on computational prediction could provide broad ideas and be a useful supplement for the mechanism exploration of TCM. In addition, the new potential lead compounds screened in our study, for example, 11-hydroxyrankinidine, may provide a rational direction for future drug discovery and development. With the development of monomer purification technology (Su et al., 2011), it will be hopefully available to identify the anti-NPP effect. What should also be stressed here is that, before experimental validation, network pharmacology prediction could serve as promising, rapid, and cost-effective strategies during the drug discovery and development process in the future.

## Conclusion

This study revealed the underlying pharmacological mechanisms of GEB on NPP based on network pharmacology and experimental evidence. Forty-seven active alkaloids might play a synergistic role in the treatment of NPP, and 11-hydroxyrankinidin had excellent stability in the active site pocket of EGFR, JAK1, and AKT1, the core targets in network pharmacology. Meanwhile, GEB participates in the regulation of 73 pathways including neuroactive ligand-receptor interaction in the pathogenesis of NPP concentrated mainly on phosphorylation reactions and nitric oxide synthesis processes. Experimental evidence also proved that GEB may regulate the expression of EGFR and JAK1 after the formation of ligand-receptor complexes. In the future, network pharmacology based on computational prediction may provide broad ideas for TCM mechanism exploration as well as cost-effective strategies during the drug discovery and development process.

## Data Availability

The raw data supporting the conclusion of this article will be made available by the authors, without undue reservation.
